# 非小细胞肺癌中EML4-ALK融合及临床治疗进展

**DOI:** 10.3779/j.issn.1009-3419.2011.05.10

**Published:** 2011-05-20

**Authors:** 荻 吴, 鸿 于

**Affiliations:** 1 130012 长春，吉林省肿瘤医院肿瘤内科 Department of Medical Oncology, Jilin Province Tumor Hospital, Changchun 130012, China; 2 130012 长春，吉林省肿瘤防治研究所细胞生物研究室 Cell Biology Laboratory, Jilin Province Tumor Institute, Changchun 130012, China

肺癌的死亡率居恶性肿瘤的首位。其中，非小细胞肺癌（non-small cell lung cancer, NSCLC）约占80%，被确诊时多为晚期。传统的化疗能改善近期疗效，对生存期的改善有限，故中位生存时间为12个月左右，预后差^[1]^。近年来，分子靶向药物治疗正在成为肺癌治疗的热点，如10%-30%的NSCLC患者携带活化的表皮生长因子受体（epidermal growth factor receptor, *EGFR*）基因突变，70%以上的*EGFR*突变患者对小分子酪氨酸激酶抑制剂（tyrosine kinase inhibitors, TKIs）高度敏感，有明显的生存获益。因此，发现和确认肺癌患者的分子亚型，寻找有效的分子靶向治疗靶点是当前需要迫切解决的问题^[2]^。

## 1 EML4-ALK的发现及其结构特点

最近发现一种新的癌基因，棘皮动物微管相关蛋白样4-间变性淋巴瘤激酶（echinoderm microtubuleassociated protein-like 4-anaplastic lymphoma kinase, EML4-ALK）参与了NSCLC的发生过程。EML4是棘皮动物微管相关蛋白样蛋白质家族成员^[3]^，其结构组成包含一个N-端基本区，一个疏水的棘皮动物微管相关蛋白样蛋白（hydrophobic echinoderm microtubule-associated protein-like protein, HELP）域和WD重复区。ALK首先在间变性大细胞淋巴瘤中被发现，作为一个核磷蛋白（nucleophosmin, NMP）的融合者，伴随一个t（2; 5）染色体重排^[4]^。EML4和ALK都位于2号染色体短臂上（2p21和2p23，间隔12个碱基），方向相反，通过染色体片段倒位产生了*EML4-ALK*融合基因。

2007年，Soda等^[5]^首次报道从1例62岁吸烟的肺腺癌患者肿瘤组织中扩增出由3, 926 bp组成的DNA片段，编码1, 059个氨基酸组成的蛋白质。蛋白质的氨基端部分（残基1-496）被鉴定为人*EML4*基因的一部分，而羧基端部分（残基497-1, 059）是人*ALK*基因的胞内域，表明该cDNA是EML4和ALK的融合产物。随后的研究^[6]^发现，*EML4-ALK*融合基因有多个亚型，所有的亚型都含有ALK胞内酪氨酸激酶结构域，始于编码外显子20的位置；EML4在融合基因中以可变的截断的形式存在，其WD重复区的数目在各亚型中不同。

涉及到ALK位点的其它染色体易位，如NPM-ALK已在淋巴瘤和炎性肌纤维母细胞瘤等肿瘤中被发现^[7]^。ALK的融合点在大多数这种嵌合的酪氨酸激酶中是保守的，包括EML4-ALK。这些融合蛋白的氨基端部分负责蛋白的寡聚化，导致组成性激活ALK激酶和异常活化下游信号，包括Akt、STAT3和细胞外信号调节激酶1/2等。正是这种细胞信号转导通路的异常活化，赋予了此类肿瘤细胞生长、增殖、抗凋亡等表型的改变，从而改变其生物学行为。

## 2 EML4-ALK亚型的鉴定

Soda等^[5]^发现EML4外显子13下游的内含子13在大约3.6 kb处中断，反转连接到ALK外显子21上游297 bp处，产生了EML4-ALK的亚型1。将表达EML4-ALK的质粒导入到小鼠3T3成纤维细胞中，证明EML4-ALK具有恶性转化活性。他们检测了33例NSCLC标本中EML4-ALK的融合频率，发现3例（9.1%）存在EML4-ALK亚型1；6例（18.2%）存在EGFR外显子19的缺失或替换，但是均没有*EML4-ALK*融合基因；*KRAS*突变患者也没有EML4-ALK融合。他们又在一组42例NSCLC患者中筛选EML4-ALK融合，结果发现在2例肺腺癌患者中存在其它EML4-ALK亚型，EML4在外显子20下游545 bp位置被中断，并融合到ALK的外显子21上游232 bp处（亚型2）。

2008年有研究^[8]^在2例NSCLC患者中发现并鉴定了EML-ALK亚型3a和3b。EML4-ALK亚型3a和3b都显示出不低于亚型1和2的转化活性和激酶活性。进一步研究EML4-ALK激活的细胞内信号通路，用荧光素酶报告基因连接*Fos*、*Myc*、*Bcl-XL*、*NF-κB*和*GAS*（STAT-1和STAT3的靶点）基因的启动子，然后将连接产物和一个表达EML4-ALK亚型3的质粒共同导入到HEK293细胞中，结果表明EML4-ALK亚型3b激活*Fos*和*Myc*基因的启动子；相反，虽然STAT3已被证明是一个NPM-ALK融合蛋白的下游目标，EML4-ALK却没有激活GAS，表明STAT3不太可能是EML4-ALK的主要靶标。EML4-ALK没有激活*Bcl-XL*基因的启动子，诱导一个弱的但是明显增强的NF-κB结合序列的激活^[3]^。

为了更灵敏地检测EML4-ALK融合，Takeuchi等^[9]^建立了一个多重逆转录PCR系统捕获两个基因间所有可能的融合，又确认了2个新的融合亚型，其中EML4外显子14（亚型4，带有额外的11个核苷酸，来源不明，反转连接到ALK的外显子20的第50位核苷酸）或外显子2（亚型5a和5b，5b的EML4的外显子2连接到ALK的外显子20，并插入了来自19内含子的117 bp）连接到ALK外显子20。EML4-ALK的新形式表现出明显的致癌活性。

与此同时，Koivunen等^[10]^筛选了305例NSCLC标本和83个NSCLC细胞系。首次确定了在白种人NSCLC患者和NSCLC细胞系中EML4-ALK的融合频率。305例中有8例（3%）存在*EML4-ALK*融合基因，其中2例为新亚型（亚型4），是EML4的外显子15与ALK的外显子20融合的产物。在167例韩国患者中有6例携带*EML4-ALK*融合基因（3.6%），138例美国患者中有2例（1.5%）携带*EML4-ALK*融合基因。这8例患者均没有*KRAS*和*BRAF*突变，但有1例患者有*EGFR*突变。所有携带EML4-ALK的肿瘤和细胞系都是腺癌。EML4-ALK在NSCLC患者中更频繁，在女性中（4%）比在男性（2%）中更高。不吸烟或轻度吸烟者高于吸烟患者（6% *vs* 1%, *P*=0.049）。另外，在83个NSCLC细胞系中发现有3个细胞系（3.6%）中存在*EML4-ALK*融合基因。其中H3122表达EML4-ALK的亚型1；H2228表达亚型3a和3b。

Takeuchi等^[11]^为了检测ALK融合，建立了一种插入式抗体强化聚合物（intercalated antibody-enhanced polymer, iAEP）方法，在主要的ALK抗体和葡聚糖聚合物检测试剂之间合并一个插入抗体，敏感性较高。在免疫组化介导的EML4-ALK检测中，使用iAEP，除了在所有的NSCLC样本中发现了已知的EML4-ALK阳性肿瘤外，又发现了新的EML4-ALK亚型（亚型6，EML4的外显子13融合到ALK的外显子20的上游的69 bp的位置；亚型7，EML4的外显子14融合到ALK的外显子20的核苷酸13）和一个新的*ALK*融合基因*KIF5B-ALK*。

目前在NSCLC及包括乳腺癌和大肠癌在内的其它肿瘤中已发现并确认了11种以上的EML4-ALK亚型（图 1）^[12]^，发生不同位点融合的原因及物质基础尚不明了，因此其发生机制引起广泛的关注。它们的转化活性和激酶活性也存在差异，这种差异在肿瘤发生过程中起到什么作用需要进一步研究阐释。不同亚型对ALK抑制剂的应答可能也会不同，值得我们在体内外研究中深入探讨。

**1:**
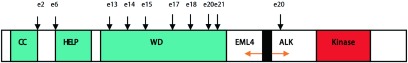
EML4-ALK融合基因结构示意图。EML4-ALK融合发生在ALK外显子20附近，在结构上能与之融合的EML4的相应的外显子的位置如箭头所示。CC：coiled-coil域；HELP：疏水的EMAP (棘皮动物微管-相关蛋白)样蛋白域；WD：WD重复区；Kinase：ALK的酪氨酸激酶域 Structure of EML4-ALK fusion gene. The corresponding positions of exons(e) of EML4 can be fused in-frame to exon 20 of ALK are indicated by arrows. CC, coiled-coil domain; HELP, hydrophobic EMAP (echinoderm microtubule-associated protein) like protein domain; WD, WD repeats; Kinase, Tyrosine kinase domain of ALK

## 3 亚裔人群*EML4-ALK*基因融合特点及临床特征

2009年Wong等^[13]^报道了中国NSCLC患者EML4-ALK的融合频率、基因表达谱和临床病理特征。在266例手术切除的原发NSCLC标本（包括腺癌、淋巴上皮样癌、鳞状细胞癌、粘液表皮样癌、腺鳞癌）和24个肺癌细胞系中（包括18个NSCLC细胞系、1个小细胞癌细胞系和5个自行建立的NSCLC细胞系^[14]^）研究了EML4-ALK的表达情况。结果表明，这些NSCLC标本中有13例（4.9%）表达EML4-ALK，其中8例表达亚型3（3a和3b）；2例表达亚型1；2例表达亚型2，还有1例标本表达新的EML4-ALK亚型（亚型5，涉及EML4的外显子18）。EML4-ALK表达与不吸烟有关（*P*=0.009）。含融合基因的肿瘤*EGFR*和*KRAS*基因呈野生型。EML4-ALK阳性的腺癌患者平均年龄更低（*P*=0.018）。24个细胞系都不表达EML4-ALK。与以前的报道不一致，该项研究没有观察到携带融合基因的患者在性别、肿瘤病理类型、肿瘤分化程度、肿瘤大小或病理发展阶段之间存在明显的相关性。其原因可能与观察病例之间的差异、NSCLC亚型和相对小的样本量有关。

2010年张绪超等^[15]^报道一组103例NSCLC患者中*EML4-ALK*融合基因的频率，确定12例（11.6%）表达EML4-ALK。1例表达亚型5（E2; A20, 408 bp）；3例表达亚型9（E18; A20, 2, 256 bp）；4例表达亚型1（E13;A20, 1, 689 bp）；1例表达亚型2（E20; A20, 2, 445 bp）；3例表达亚型3a/b（E6；A20或E6 ins33；A20，867 bp和900 bp）。EML4-ALK在腺癌患者中的表达频率为16.13%（10/62），在不吸烟者中为19.23%（10/52），在缺乏*EGFR*和*KRAS*突变的腺癌患者中为42.80%（9/21）。EML4-ALK融合与非吸烟者（*P*=0.03），较年轻的发病年龄（*P*=0.03），和无*EGFR*/*KRAS*突变的腺癌（*P*=0.04）相关。*ALK*基因重排和EML4-ALK mRNA和蛋白表达水平之间的相关性一直存在争议。该研究表明，EML4-ALK融合似乎是与ALK mRNA的表达水平紧密相关，因为在EML-ALK融合阳性的肿瘤中ALK mRNA表达水平更高（相比阴性肿瘤，*P*=0.001, 8），相反，EML4的表达在群体中没有差异。值得注意的是，他们也发现1例女性不吸烟腺癌患者的样本中同时存在*EGFR*突变（外显子19缺失）和EML4-ALK融合基因（亚型3b），提示这两个基因组变化在癌症的发生和发展中的作用还需要更加深入的研究^[10]^。

复旦大学肿瘤医院进行了一项对东亚非吸烟肺腺癌中主要复发癌基因突变的研究^[16]^。52例不吸烟肺腺癌患者的手术切除标本，同时检测*EGFR*、*KRAS*、*NRAS*、*HRAS*、*HER2*、*BRAF*、*ALK*、*PIK3CA*、*TP53*和*LKB1*突变。结果表明，41例肿瘤包含*EGFR*突变；3例携带EML4-ALK融合；2例有HER2插入；1例*KRAS*突变。所有突变相互排斥。未检测到*BRAF*、*NRAS*、*HRAS*或*LKB1*突变，15例*TP53*突变，4例包含伴有*EGFR*突变的*PIK3CA*突变，针对目前存在的分别以*EGFR*、*HER2*和*ALK*突变为靶标的药物，这个结果表明前瞻性突变检测对个体化的靶向治疗具有重要意义。

随着对EML4-ALK研究的深入，引出的新问题越来越多，尤其是一些问题与研究者最初的设想背道而驰。*EML4-ALK*基因融合与*EGFR*和*KRAS*基因突变是排斥的，还是各自独立发生的？*EML4-ALK*基因融合在正常组织中表达，在肺癌以外的其它肿瘤组织中表达，打破了其仅是NSCLC中的特征性基因改变这一最初的认识，使它在肿瘤的发生、发展过程中到底发挥何种作用变得越来越复杂^[17]^。这些问题影响EML4-ALK重排在NSCLC的发病，诊断和分子靶向治疗中的作用。

## 4 ALK抑制剂的应用

*ALK*基因编码一种受体酪氨酸激酶（receptor tyrosine kinase, RTK）。活化的ALK通过激活PI3K/Akt信号和MAPK信号通路的下游信号参与抑制细胞凋亡、促进细胞增殖^[18]^。深入研究ALK激酶活性和患者样本中下游信号激酶的激活之间的相关性特别重要。

EML4-ALK可以作为一个潜在的治疗靶点。单独或组合应用ALK激酶抑制剂对携带EML4-ALK的NSCLC患者可能是有效的临床治疗手段。Soda等^[19]^研究了一种ALK抑制剂（WHI-P154）对EML4-ALK1阳性NSCLC的作用。结果表明WHI-P154显著抑制表达EML4-ALK的BA/F3细胞的生长，在10 μmol/L浓度时迅速导致这些细胞死亡。WHI-P154以一种浓度依赖方式抑制EML4-ALK的酪氨酸磷酸化。将EML4-ALK亚型3b导入到IL-3依赖的BA/F3细胞中，然后将细胞暴露于另一个ALK激酶抑制剂（2, 4-pyrimidinediamine）。结果发现BA/F3细胞迅速死亡。2, 4-Pyrimidinediamine以一种浓度依赖方式抑制了EML4-ALK的激酶活性。人肺癌细胞株NCI-H2228表达EML4-ALK的亚型3a和3b，2, 4-pyrimidinediamine抑制NCI-H2228细胞的生长也是以一种浓度依赖的方式。

Galkin等^[20]^评价了TAE684，一个高潜力的ALK激酶抑制剂，对携带EML4-ALK的细胞生长的影响。结果表明在3个表达EML4-ALK的细胞系中，TAE684只是明显抑制H3122细胞系的生长并导致细胞凋亡，而且只有H3122细胞系中存在明显的下游信号蛋白Akt和ERK1/2磷酸化的抑制。有趣的是，该研究发现TAE684只是导致STAT3磷酸化程度降至最小，由于STAT3对NPM-ALK介导的淋巴瘤生成是关键的，因此STAT3在携带EML4-ALK的NSCLC中到底有何作用，携带NPM-ALK和EML4-ALK的肿瘤之间到底何种信号不同尚需深入研究。

Kwak等^[21]^总结了一项涉及NSCLC患者的Ⅰ期试验研究。1, 500例NSCLC患者中发现82例（5.5%）携带一个ALK重排，这些重排并不都是EML4-ALK，表明还有其它的ALK融合存在，如TFG-ALK和KIF5B-ALK。一种新型潜在的ALK激酶抑制剂：crizotinib（PF-02341066），被应用于该试验。研究者们观察到57%的应答率和8周内87%的疾病控制率^[22]^。平均治疗时间为6.4个月，27例疾病稳定，46例部分缓解和1例完全缓解。所有患者MET阴性（另一个crizotinib靶点），表明治疗是通过抑制ALK实现的。

除了crizotinib，其它TKI已被证明具有明显的体内抗肿瘤活性。不幸的是，对相应的TKI来说，总有一小部分肿瘤从开始治疗或在发生初级反应后就存在耐药性。Choi等^[23]^报道了1例EML4-ALK阳性的NSCLC病例，应用crizotinib成功治疗5个月后发生耐药。患者28岁，男性，无吸烟史，诊断为肺腺癌，未携带*EGFR*突变，接受了常规化疗。然而，经过6个疗程的治疗后肿瘤进展，检测发现肿瘤表达EML4-ALK的亚型1。在口服crizotinib治疗后1周，患者症状明显好转。然而5个月后，肿瘤突然开始恢复增长，导致胸腔积液的迅速增长和双侧肺部肿瘤进展。分析患者的胸腔积液样本，发现在EML4-ALK激酶域存在两个突变（野生型ALK的cDNA核苷酸4, 374和4, 493，分别发生了G→A和C→A的改变，频率为41.8%和14.0%），每个都能赋予对ALK抑制剂的抗性。没有发现任何EML4-ALK的cDNA同时携带这两个突变，因此研究者认为每个突变是独立发生的。因为无治疗前基线数据，所以不知道抗性克隆属于原发的还是继发的。这例患者出现的对crizotinib耐药突变表明，还需要其它ALK抑制剂靶向治疗crizotinib耐药的*EML4-ALK*基因融合病例。在这方面已经取得一些进展，这类药物也正在研发过程中，包括新型ALK抑制剂^[24]^。

## 5 展望未来

Shaw等^[25]^研究证明EML4-ALK阳性与EGFR TKI耐药相关。此外还发现，与无*EML4-ALK*基因融合或EGFR野生型肿瘤患者相比，EML4-ALK阳性的肿瘤患者对以铂类为基础的联合化疗显示出相似的敏感性，整体存活率无差异。

目前看来，亚裔人群*EML4-ALK*基因融合发生率略高于高加索人群，同样与不吸烟、较年轻的发病年龄相关。与性别、病理类型、肿瘤分化程度等因素的关系尚不明朗^[26-31]^。

许多临床相关问题没有解决，如是否ALK能单独驱动肿瘤的发生、发展，或在肿瘤发展过程中是否有特定分子协同ALK发挥其功能。此外，由于各种ALK融合蛋白有不同的亚细胞定位（可能依赖于融合参与蛋白的性质），它们的下游信号通路可能是不同的^[32]^。

首要的问题是寻找到最佳途径以检测ALK融合的存在。现行检测方法有多种：包括免疫组化、荧光原位杂交和RT-PCR。但是现在还没有一种公认的简便易行的标准检测手段^[33]^。张绪超等^[15]^建立了一种高度敏感的PCR诊断方法可能有广泛的应用前景。未来的工作需要确定标准的检测方法，以便在肺癌中常规地诊断性检测ALK融合。

其次，我们如何找到合适的患者进行治疗呢？理想的情况是同时检测三种目前最常见突变——*KRAS*、*EGFR*和*ALK*融合。现在提出了一种逐步交替方法来检测肺腺癌，首先检测*KRAS*突变（白人发生率为15%-30%，东亚患者的突变率约8%）。如果*KRAS*突变阳性，则不需要进一步的分子检测（*KRAS*突变的肿瘤对ALK抑制剂的敏感性有待深入研究）。如果*KRAS*突变是阴性的，再检测*EGFR*突变（白人发生率约10%，亚洲患者约30%）。如果是EGFR突变阳性，建议使用EGFR TKI治疗。如果是阴性的，肿瘤再被评估ALK易位。如果ALK是阳性的，治疗将使用ALK抑制剂，如果是阴性，这些三重阴性的肿瘤应考虑分析罕见的突变（如*BRAF*、*MEK1*、*AKT1*、*PIK3CA*等）和/或深入研究找出新的驱动突变^[6]^。

由于治疗效果可能是个变量，在通路激活机制的基础上，出现抗药性几乎是可以肯定的。目前缺乏对ALK结构信息的研究，包括ALK胞外区独特的组织结构和ALK胞内域的晶体结构，还要考虑ALK的结晶过程^[34]^。研究ALK激酶域的晶体结构^[35]^，发现L1196M残基突变可能会阻止crizotinib结合到ALK。但*C1156Y*突变的作用还不清楚，因为它似乎不太可能直接影响crizotinib结合到ALK。在体外，*C1156Y*突变对crizotinib耐药的影响不像在体内那么强，表明在细胞中该突变可能需要与其它因素相互作用，才会有更高的药物敏感性。需要详细地分析揭开ALK融合蛋白中存在的大量差异，以确定将来的干预治疗靶点^[36]^。

包括激酶抑制剂在内的抗肿瘤药物的一个主要问题是毒性作用。Kwak等^[37]^和Butrynski等^[38]^均报道口服crizotinib（250 mg/2次/天）在患者中仅发生二级不良反应，最常见的不良反应为恶心、呕吐、乏力、腹泻。

总之，上述研究为成功治疗ALK阳性的恶性肿瘤提供了一个乐观的前景。在涉及ALK的癌症患者群体中，一个标准的基因分型方法对制定更加个体化的治疗方案将是非常重要的。将来的crizotinib和其它ALK抑制剂的临床研究将会告诉我们是否它们是战胜癌症的新生力量^[36]^。

除了使用ALK抑制剂，发展一种同时抑制ALK信号通路多个靶点的治疗策略也是至关重要的，还应发展针对ALK的单克隆抗体治疗。此外，是否单独使用ALK抑制剂或与其它激酶抑制剂组合使用才能有效地抑制肿瘤细胞生长和诱导凋亡呢？

显然，采用靶向治疗，我们将需要筛查肿瘤的大量的突变，完成总的基因组分析。需要正确处理组织来完成这样的分析。随着全球多机构和多学科的通力合作，将来应该有可能对肿瘤精确分子分型以确定合适的治疗靶点，更有可能在治疗前就预测出耐药亚群^[39]^。
